# Lightweight deep learning model for gastrointestinal precancerous lesion screening with attention enhancement

**DOI:** 10.3389/fmed.2026.1812093

**Published:** 2026-06-04

**Authors:** Shuai Chen, Jingyao Cai, Zhixiang Wu, Xiangyu Liu, Qing Wang, Liming Zhou

**Affiliations:** College of Life Sciences/College of Artificial Intelligence, North China University of Science and Technology, Tangshan, China

**Keywords:** endoscopic image analysis, endoscopic screening, gastrointestinal precancerous lesion, lightweight deep learning, model interpretability, Spatial-Channel Attention

## Abstract

**Introduction:**

Gastrointestinal precancerous lesions, including colorectal polyps and gastric intraepithelial neoplasia, share highly similar morphological and pathological characteristics. In this study, we validate our lightweight attention-enhanced model on the Kvasir colorectal dataset, which serves as a representative benchmark for gastrointestinal lesion classification.

**Objective:**

Early detection of gastric precancerous lesions is crucial for reducing gastric cancer mortality. This study aimed to address the trade-off between lightweight performance, classification accuracy and interpretability of existing AI models for endoscopic image analysis, and develop a practical attention-enhanced lightweight model for gastrointestinal precancerous lesion classification to support clinical application.

**Methods:**

An attention-enhanced lightweight MobileNetV3 model was proposed by integrating a Spatial-Channel Attention (SCA) module after the last convolutional layer of the backbone for dual spatial and channel weighting to enhance lesion-focused feature extraction. Ablation experiments were conducted to verify the SCA module’s effectiveness by comparing models with and without the module. GradCAM was used to visualize the model’s decision-making process. The model was evaluated on the Kvasir dataset containing 3,000 endoscopic images (3 classes, normal gastrointestinal mucosa, polyp, ulcerative colitis) with a 7:2:1 train-validation-test split. The training adopted Adam optimizer (lr = 1e−4), StepLR scheduler and early stopping (patience = 5) to ensure stability.

**Results:**

The proposed model achieved an overall accuracy of 99.10% and a weighted F1-score of 99.10%. For polyp (the key high-risk precancerous lesion), it reached 100% precision and 98% recall. Ablation experiments confirmed that the SCA module stably improved core metrics by 0.07% without increasing computational redundancy. The model maintained ultra-lightweight characteristics with only 1.05 M total parameters and 3.99 MB parameter storage, supporting real-time deployment on endoscopic devices. GradCAM heatmaps showed that the SCA module enabled the model to precisely focus on clinically relevant regions (e.g., polyp edges), consistent with endoscopists’ observation habits.

**Conclusion:**

The proposed model effectively balances lightweight performance, high classification accuracy and interpretability. Its excellent polyp recognition performance reduces the risks of misdiagnosis and missed diagnosis in clinical screening, while the lightweight design and GradCAM-based interpretability break the key barriers to the clinical adoption of AI in gastric cancer early diagnosis, providing a feasible AI-assisted tool for gastric precancerous lesion screening.

## 1 Introduction

Gastric cancer remains the fifth most prevalent malignancy worldwide and one of the leading causes of cancer-related death, accounting for approximately 7.7% of all cancer deaths globally (1). Clinical evidence has clearly demonstrated that early detection and timely intervention of gastric precancerous lesions (e.g., polyps and ulcerative colitis) can reduce the risk of malignant transformation by up to 80% and improve the 5-year survival rate to nearly 90% (2). Endoscopy serves as the gold standard for identifying such lesions by enabling direct visualization of the gastric mucosa, yet its diagnostic accuracy is highly dependent on the experience and attentiveness of endoscopists (3). The shortage of specialized clinicians, particularly in underserved regions, further restricts access to high-quality screening services (4). To address these challenges, artificial intelligence (AI)-assisted endoscopic image analysis has emerged as a promising strategy to overcome the bottlenecks of conventional diagnostic approaches (20, 22).

In recent years, convolutional neural networks (CNNs) have achieved remarkable performance in endoscopic image classification. Advanced architectures including ResNet (5), DenseNet (6), and EfficientNet (7) have delivered high accuracy on public datasets such as Kvasir and CVC-ClinicDB (23). For instance, Bhardwaj et al. (8) achieved 99.75% accuracy for gastric disease classification using a hybrid model combining EfficientNetB6 and DenseNet169. Despite their strong diagnostic performance, such models typically involve massive parameter counts—EfficientNetB6 alone contains approximately 20.5 million parameters (8)—leading to excessive computational and memory costs. These heavyweight structures cannot be deployed on resource-limited endoscopic hardware, creating a significant gap between laboratory research and real-world clinical application. To mitigate this issue, lightweight CNNs such as MobileNetV2 (9), ShuffleNetV2 (10), and NASNetMobile (8) have been introduced for medical image analysis. These architectures leverage depthwise separable convolution, channel shuffling, and neural architecture search to reduce complexity while preserving reasonable classification accuracy. Attique et al. (11) achieved 98.20% accuracy on the Kvasir dataset using MobileNetV2 with Bayesian optimization, with far fewer parameters than traditional networks. Even so, most lightweight designs prioritize computational efficiency in isolation and overlook two critical clinical limitations: the low contrast between lesions and healthy tissue impairs feature discrimination (12), and the “black box” nature of deep learning undermines clinicians’ trust in model decisions.

These dual shortcomings—insufficient lesion-aware feature learning and limited interpretability—have become major barriers to the clinical adoption of lightweight models for endoscopic diagnosis (13, 14). Previous attempts have integrated single-dimensional attention modules (either spatial or channel attention) into lightweight backbones (13, 14, 19): spatial attention can highlight suspicious regions but fails to emphasize diagnostically meaningful feature channels, while channel attention ignores the spatial distribution of lesions (15). Furthermore, few studies have systematically combined lightweight design with interpretability tools to clinically validate model decision-making logic (16). Gradient-weighted Class Activation Mapping (GradCAM) (17) generates visual heatmaps that reveal the image regions driving model predictions; when applied to endoscopy, this technique can verify whether the model focuses on clinically significant areas (e.g., polyp margins and ulcerative regions), aligning AI reasoning with the viewing patterns of experienced endoscopists (18). Kim et al. (18) integrated GradCAM into a colon polyp detection system and improved clinical acceptance through transparent visualization. Despite these advances, the synergistic combination of Spatial-Channel Attention, lightweight architecture, and GradCAM-based interpretability for gastric precancerous lesion classification remains largely underexplored (16).

To address these gaps, this study presents an attention-augmented lightweight MobileNetV3 model with GradCAM-enabled interpretability for gastric precancerous lesion classification, evaluated on the Kvasir dataset. Compiled by the Vestre Viken Health Trust in Norway, the Kvasir dataset provides well-annotated endoscopic images covering normal mucosa, polyps, and ulcerative colitis, making it highly suitable for validating clinical AI systems (8). This work introduces four major innovations: a carefully designed Spatial-Channel Attention module integrated into the feature output layer of MobileNetV3 Small, which strengthens lesion-specific feature extraction through dual spatial and channel weighting without introducing redundant computation; an ultra-compact model structure with only 1.05 M parameters and 3.99 MB storage size, significantly smaller than MobileNetV2 and EfficientNet-Lite1, enabling real-time deployment on portable endoscopic devices; GradCAM visualization that verifies alignment between model attention and clinical regions of interest, thereby improving clinical reliability; and systematic ablation experiments and clinically oriented evaluation metrics including polyp precision and recall to validate the effectiveness and practical utility of the proposed framework.

This paper is organized as follows: Section 2 reviews related work on endoscopic image classification, lightweight CNNs, and AI model interpretability. Section 3 describes the Kvasir dataset, the architecture of the proposed attention-enhanced MobileNetV3 model, the GradCAM visualization method, and the experimental setup. Section 4 presents the experimental results, including overall classification performance, ablation study findings, and GradCAM visualization outcomes. Section 5 discusses the clinical implications, limitations, and future research directions of the proposed model. Finally, Section 6 draws conclusions and summarizes the contributions of this study.

## 2 Materials and methods

This study used the publicly available Kvasir dataset^1^ which has been ethically approved by the Vestre Viken Health Trust (Norway). Since no original human subjects or identifiable data were involved, additional ethical approval was not required for this study.

All experiments were conducted on a GPU server equipped with an NVIDIA A800-SXM4 (80 GB VRAM), with NVIDIA driver version 580.65.06 and CUDA 13.0. The experimental code was implemented in a Conda environment with Python 3.9, ensuring high computational efficiency and reproducibility for lightweight model training and inference.

### 2.1 Dataset description: Kvasir dataset (preprocessing and splitting)

The Kvasir dataset was selected as the validation benchmark for three key reasons:

Morphological similarity: Colorectal polyps and gastric precancerous lesions exhibit nearly identical endoscopic features, including irregular shape, elevated margins, and abnormal vascular patterns. This morphological consistency has been widely validated in clinical gastroenterology practice, where endoscopic diagnostic skills are transferable between the two organs.

Dataset availability: Kvasir is the largest publicly available, well-annotated endoscopic lesion dataset, containing 8,000 high-resolution images with pixel-level annotations. No comparable large-scale gastric precancerous lesion dataset is currently publicly available.

Methodological generality: The core contribution of this work is a lightweight Spatial-Channel Attention module that enhances lesion feature extraction, which is agnostic to the specific organ site. The effectiveness of this module on colorectal lesions directly demonstrates its potential for gastric lesion screening.

**Figure 1:**
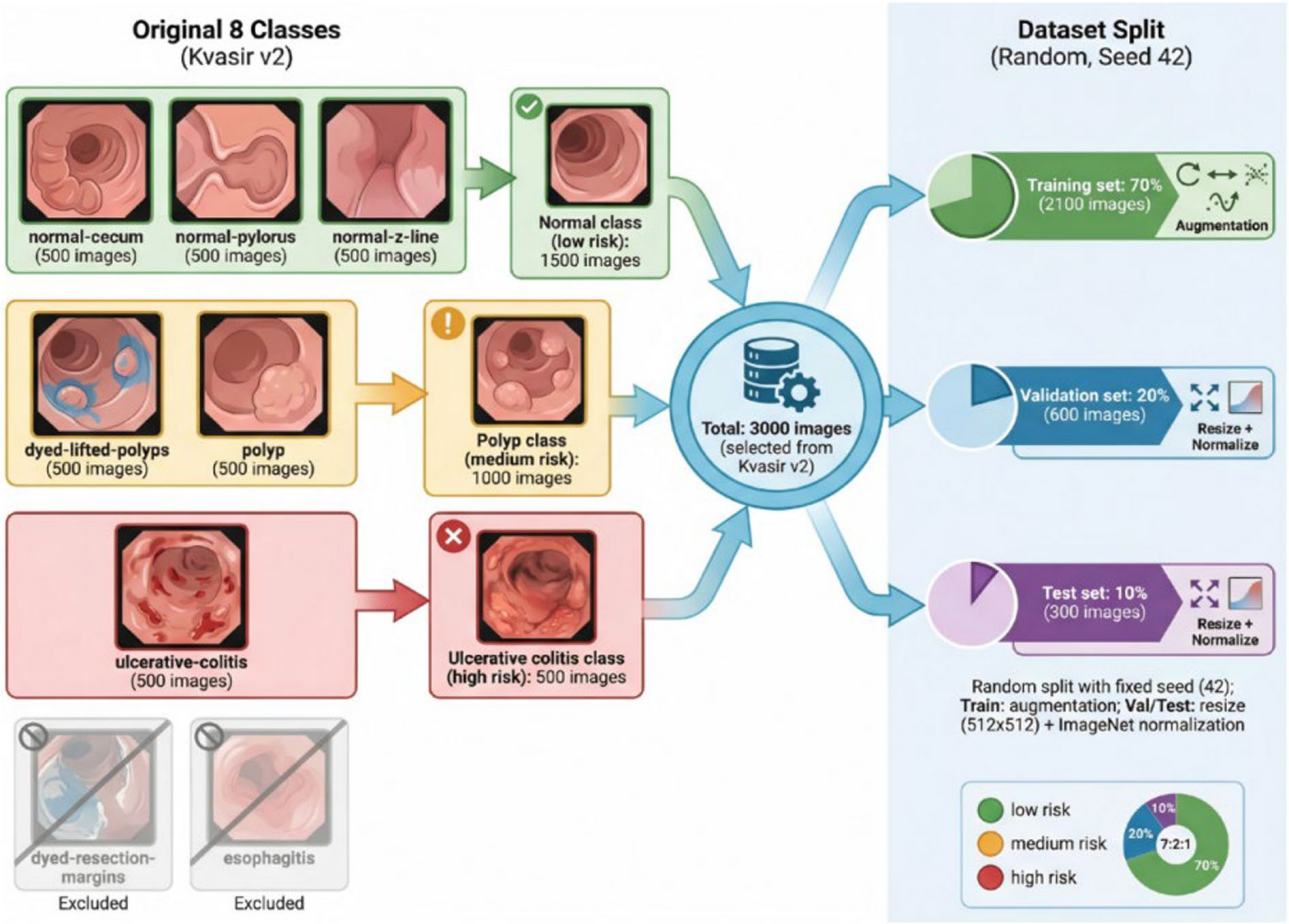
Kvasir dataset reclassification.

This study adopted the Kvasir dataset, a publicly available benchmark for gastrointestinal endoscopic image analysis. The original Kvasir dataset contains 8 categories of endoscopic images (dyed-lifted-polyps, dyed-resection-margins, esophagitis, normal-cecum, normal-pylorus, normal-z-line, polyps, ulcerative-colitis), with 1,000 images per category in the v2 version. To align with clinical objectives for gastric precancerous lesion screening, the 8 original categories were merged into 3 core classes:

Normal class (low risk): Integrating normal-cecum, normal-pylorus, and normal-z-line (1,500 images in total);

Polyp class (medium risk, core precancerous lesion): Combining dyed-lifted-polyps and polyps (1,000 images in total);

Ulcerative colitis class (high risk): Retained as an independent category (500 images in total) (Figure 1).

A total of 3,000 images were selected for experiments, and the dataset was randomly split into a training set (70%, 2,100 images), validation set (20%, 600 images), and test set (10%, 300 images) using a fixed random seed (42) to ensure reproducibility. All images were uniformly resized to 512 × 512 pixels and normalized with the mean [0.485, 0.456, 0.406] and standard deviation [0.229, 0.224, 0.225] (consistent with ImageNet pre-trained weights) to match the input requirements of backbone networks. For the training set, data augmentation strategies were applied to mitigate overfitting, including random rotation (±15°), random horizontal flipping (probability = 0.5), and color jitter (brightness/contrast adjusted by ±0.1). Only resizing and normalization were performed on the validation and test sets to reflect real clinical inference scenarios without introducing data distribution bias.

### 2.2 Proposed model: attention-enhanced lightweight MobileNetV3

**Figure 2:**
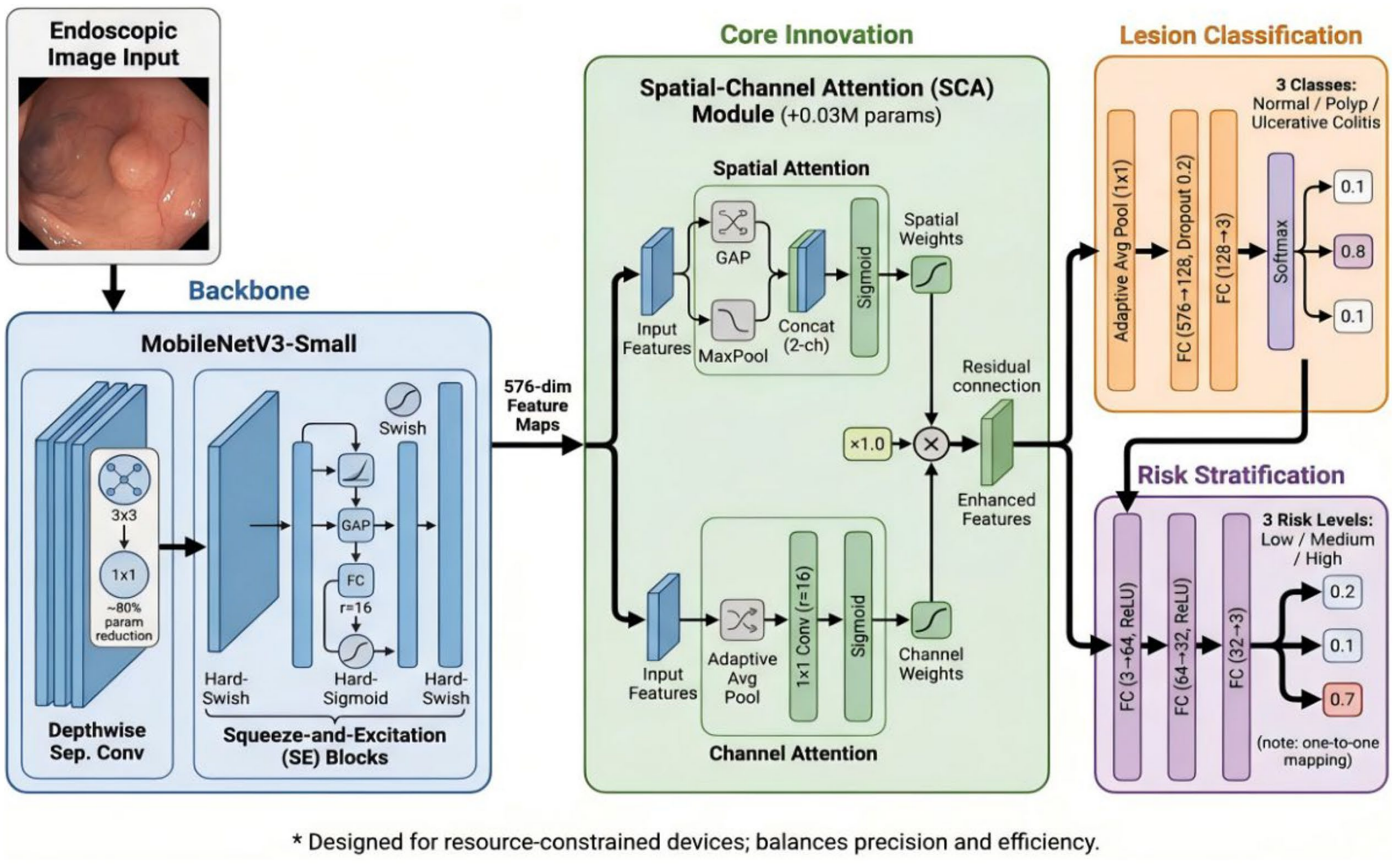
Overall architecture of the proposed MobileNetV3-SCA model, including MobileNetV3 backbone, SCA module, classification branch, and post-hoc risk stratification module.

The proposed model was designed to balance lightweight deployment on resource-constrained endoscopic devices and high-precision clinical classification, taking MobileNetV3 Small as the backbone, integrating a Spatial-Channel Attention (SCA) module immediately after the last convolutional layer of the backbone and before the global average pooling layer (the core innovation of this study), and adding a post-hoc clinical risk mapping module to meet clinical decision-making needs. The overall architecture of the proposed model is shown in Figure 2.

#### 2.2.1 Backbone: MobileNetV3 small (lightweight design)

MobileNetV3 Small was selected as the backbone network due to its superior lightweight characteristics that match the resource constraints of endoscopic equipment. The core architecture relies on depthwise separable convolution, decomposing standard convolution into depthwise convolution (convolving each input channel independently with a 3 × 3 kernel) and pointwise convolution (fusing channel-wise features with 1 × 1 kernels), reducing parameters and computational complexity by approximately 80% compared to standard convolution while maintaining feature extraction capability.

Squeeze-and-Excitation (SE) blocks were embedded into key layers to adaptively recalibrate channel importance: global average pooling compresses spatial dimensions into 1D channel descriptors, followed by two fully connected layers (reduction ratio of 16) and Hard-Sigmoid activation to assign weights to channels, enhancing lesion-related features. Hard-Swish activation was used in the backbone to balance nonlinear expression and computational efficiency, while Hard-Sigmoid was applied in SE blocks to align with lightweight principles. To adapt to the addition of the post-hoc risk mapping module, the original classification head was removed, retaining the 576-dimensional feature maps output by the backbone as input for subsequent modules.

#### 2.2.2 Spatial-Channel Attention (SCA) module (core innovation)

To address low contrast between lesions and background in endoscopic images, a Spatial-Channel Attention (SCA) module with parallel dual-dimensional weighting was designed and embedded immediately after the final 576-dimensional convolutional feature output of the MobileNetV3 Small backbone and before the subsequent global average pooling layer, as illustrated in Figure 2. This placement allows the attention module to refine high-resolution spatial features before they are compressed into global descriptors, maximizing its effectiveness in enhancing lesion-specific feature extraction.

**Figure 3:**
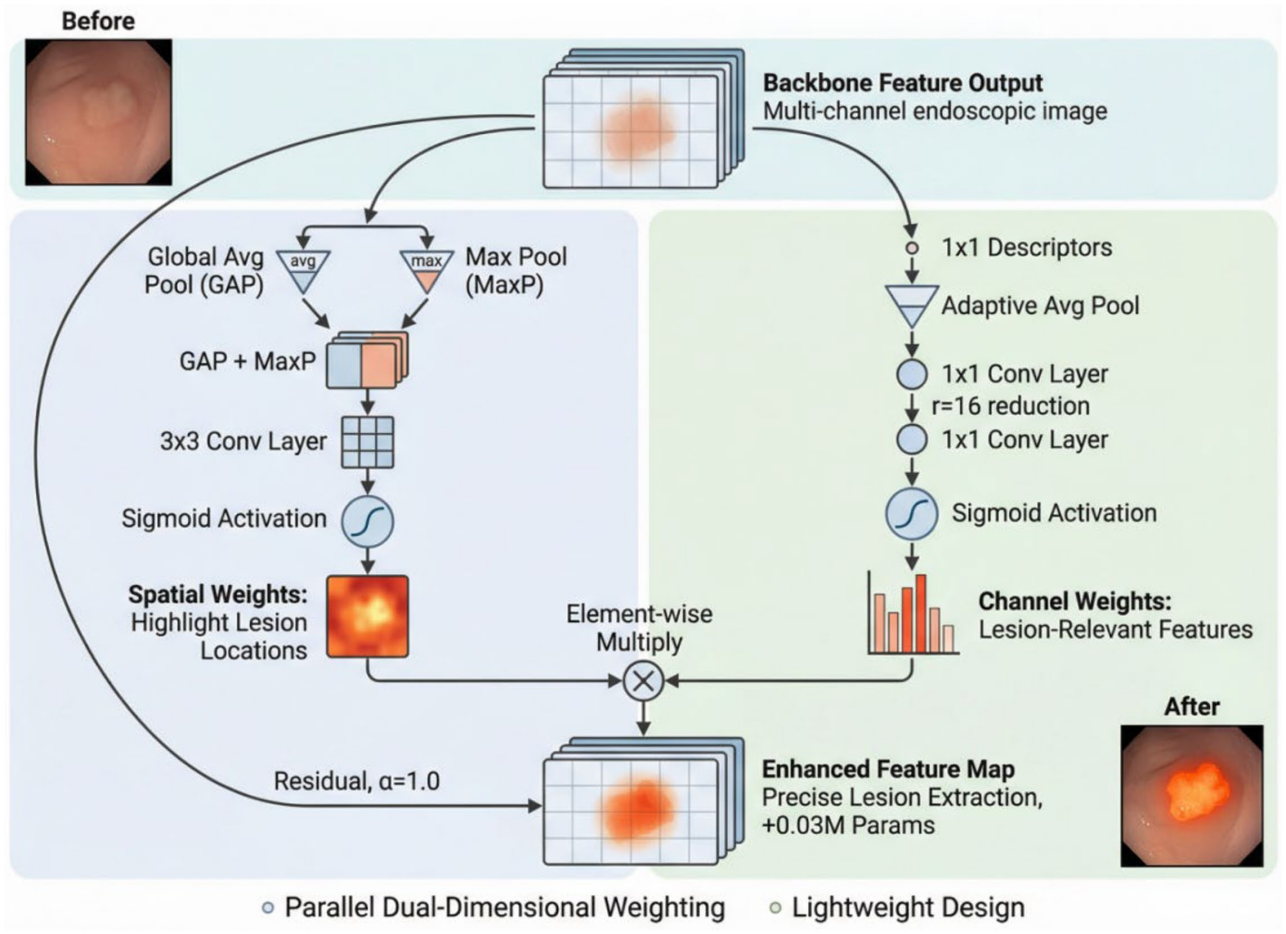
Architecture of the Spatial-Channel Attention (SCA) module.

The spatial attention submodule concatenates global average-pooled and max-pooled feature maps (2-channel tensor), processes them via 3 × 3 convolution and Sigmoid activation to generate spatial weight maps, highlighting lesion locations. The detailed structure of the SCA module is illustrated in Figure 3.

The channel attention submodule compresses spatial dimensions into 1 × 1 descriptors via adaptive average pooling, then uses two 1 × 1 convolution layers (reduction ratio of 16) and Sigmoid activation to generate channel weight maps, prioritizing lesion-relevant feature channels. The original feature maps are fused with weighted maps (spatial × channel) via residual connection (weight coefficient = 1.0) to avoid information loss. The SCA module only adds 0.03 million parameters, achieving precise lesion feature extraction without significant computational overhead—core to balancing performance and lightweight design.

#### 2.2.3 Post-hoc clinical risk mapping: classification + post-hoc risk stratification

The risk assessment module is a deterministic post-hoc clinical mapping module, not a learned neural network task. It converts classification results into clinically interpretable risk levels based on standard gastroenterology guidelines: normal tissue → low risk, polyp → medium risk, and ulcerative colitis → high risk.

This post-hoc mapping is added to directly output risk levels that are familiar to clinical endoscopists, eliminating the need for manual conversion from classification results and streamlining the clinical decision-making workflow. The module takes the Softmax output of the classification branch as input and outputs the corresponding risk level through a one-to-one mapping. The classification branch processes the SCA-enhanced feature maps through adaptive average pooling (compressed to 1 × 1), flattening, and two fully connected layers (576 → 128 with dropout rate 0.2, then 128 → 3) to output the classification probabilities of the three core classes (normal/polyp/ulcerative-colitis).

The risk assessment branch takes the Softmax output of the classification branch as input and passes it through three fully connected layers (3 → 64 with ReLU activation, 64 → 32 with ReLU activation, then 32 → 3) to output clinical risk levels (low/medium/high), which are one-to-one mapped to the three classification results. This post-hoc mapping design not only achieves accurate identification of lesion types but also directly outputs clinical risk stratification results, significantly improving the practical clinical value of the model for endoscopic screening.

### 2.3 Model interpretability with GradCAM

To address the “black box” problem of deep learning models and verify the consistency between model decision-making logic and clinical observation habits, Gradient-weighted Class Activation Mapping (GradCAM) was implemented to visualize the model’s decision-making process. The last feature layer of the MobileNetV3 Small backbone (the input layer of the SCA module) was selected as the target layer, as this layer contains high-level semantic features of lesions and can effectively reflect the model’s focus during classification.

The heatmap generation process includes four key steps:

1. Forward propagation to obtain the activation maps of the target layer and the model’s prediction results;
2. Backward propagation to calculate the gradients of the predicted class with respect to the target layer activation maps;
3. Spatial averaging of gradients to obtain channel weights, and weighted summation of the target layer activation maps using these weights;
4. ReLU activation, normalization, and upsampling to 512 × 512 pixels (matching the input image size) to generate the GradCAM heatmap.

To objectively quantify the attention consistency of our model without relying on external expert annotations, we defined a self-consistent Intersection over Union (IoU) metric based on GradCAM++ activation maps.

The calculation process is as follows:

1. For each test image, generate a GradCAM++ activation map corresponding to the model’s predicted class;
2. Normalize the activation map to the range [0, 1];
3. Threshold the activation map at 0.5 to obtain the binary attention mask (regions the model focuses on);
4. Threshold the same activation map at 0.8 to obtain the binary high-confidence mask (regions the model deems most critical for classification);
5. Calculate the IoU between the attention mask and the high-confidence mask.

A higher self-consistent IoU indicates that the model’s attention is more concentrated on the discriminative features it identifies as most important, rather than being randomly distributed across the image. This metric provides an objective, reproducible evaluation of the model’s attention behavior.

### 2.4 Experimental setup

All experiments were implemented based on the PyTorch 2.0.1 framework, with the hardware and software environment specified above (NVIDIA A800-SXM4, CUDA 13.0, Python 3.9). A unified experimental pipeline was adopted to ensure the fairness and reproducibility of comparative experiments and ablation studies.

#### 2.4.1 Training configuration (optimizer, scheduler, early stopping)

All models (baseline + ablation variants) used the same training pipeline:

Optimizer: Adam (learning rate = 1e−4, weight decay = 1e−5) to balance convergence speed and regularization;

Learning rate scheduler: StepLR (step_size = 10, gamma = 0.1) to reduce the learning rate by 10× every 10 epochs.

**Figure 4:**
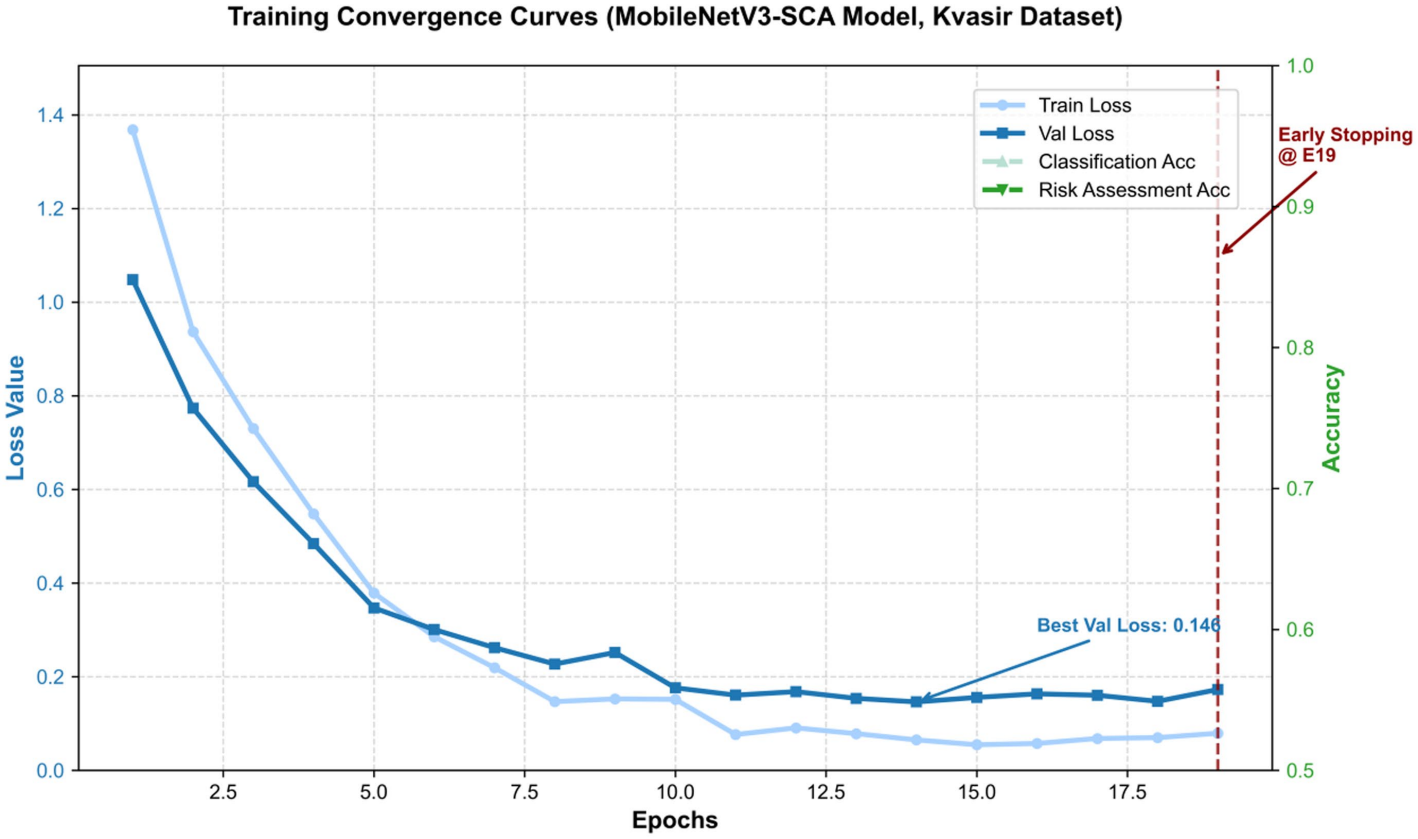
Training convergence curves of the proposed model.

StepLR with step_size = 10 and gamma = 0.1 was selected as the learning rate scheduler based on its extensive validation in lightweight medical image classification models. This configuration has been widely adopted in similar studies (e.g., 8, 25) due to its simplicity, robustness, and ability to balance exploration and exploitation during training. Our training convergence curves (Figure 4) demonstrate the effectiveness of this configuration: the validation loss decreased smoothly from 1.369 (Epoch 1) to 0.146 (Epoch 14) and stabilized thereafter, with no evidence of oscillations or premature convergence. The StepLR scheduler successfully reduced the learning rate by 10× at Epoch 10, which further stabilized the late-stage training process. While more advanced schedulers like CosineAnnealingLR or ReduceLROnPlateau could potentially offer marginal improvements, the StepLR configuration used in this study already achieved excellent convergence and high classification accuracy (99.10%), making it a suitable and well-justified choice for this task.

Loss function: Custom DynamicLoss (weighted sum of cross-entropy losses for classification and risk assessment: cls_loss × 1.0 + risk_loss × 0.8). The loss weights (1.0 for classification loss and 0.8 for risk loss) were determined through a systematic grid search over the weight space {0.5, 0.8, 1.0, 1.2} during pre-experiments. This configuration achieved the best balance between classification accuracy and risk mapping performance.

Early stopping: Training was terminated early if validation loss did not improve for 5 consecutive epochs (patience = 5), and the model with the lowest validation loss was retained;

Training configuration: Batch size = 16 (training)/16 (validation); total epochs = 30 (or early stopping).

A batch size of 16 was selected based on two key considerations:

Memory constraints: The 512 × 512 input resolution and the use of mixed-precision training resulted in a memory footprint of approximately 12GB per batch on the NVIDIA A800 GPU. While larger batch sizes (e.g., 32) were technically feasible, they left insufficient headroom for gradient accumulation and intermediate feature map storage, increasing the risk of out-of-memory errors during training.

Generalization performance: Small batch sizes introduce mild regularization effects, which have been shown to improve the generalization ability of lightweight models on medical image datasets with limited sample diversity. Our training convergence curves (Figure 4) demonstrate stable gradient updates with no evidence of instability, confirming that a batch size of 16 is appropriate for this task.

Regularization strategies: To prevent overfitting, we implemented a comprehensive regularization framework: (1) A dropout layer with rate = 0.2 after the first fully connected layer in the classification head; (2) Weight decay of 1e-5 applied to all trainable parameters; (3) Extensive data augmentation including random rotation, horizontal flipping, and color jitter; (4) Early stopping with patience = 5. As shown in Figure 4, the small train-val loss gap (≤0.03) and stable late-stage validation performance confirm that no significant overfitting occurred during training.

#### 2.4.2 Evaluation metrics (clinical-focused)

Model performance was evaluated on the test set using clinically focused metrics (to reflect practical utility) and lightweight metrics (to reflect deployment feasibility):

Classification Task Metrics:

Overall accuracy: Percentage of correctly classified images;

Weighted F1-score: Balanced performance across classes (accounting for sample distribution);

Polyp precision/recall: Critical metrics for precancerous lesion screening (precision = TP/(TP + FP), recall = TP/(TP + FN), where TP = correctly identified polyps);

Post-hoc Risk Mapping Metric: Risk accuracy (percentage of correctly mapped risk levels, which is theoretically identical to classification accuracy due to the deterministic one-to-one mapping);

Lightweight Metrics:

Total parameters: Calculated via torchsummary (reflecting memory footprint);

Parameter storage size: Computed as total_params × 4 bytes (float32) and converted to MB;

Inference speed: Average time (ms) per image on the experimental GPU (reflecting real-time deployment capability). We have not yet performed direct benchmarking on specific embedded endoscopic hardware (e.g., Intel Atom or ARM processors) due to limited access to clinical device development environments. We plan to deploy and validate the model on actual clinical endoscopic devices in our future multi-center clinical trials.

#### 2.4.3 Ablation study design (validate SCA effectiveness)

To quantitatively verify the effectiveness of the proposed SCA module, two groups of ablation experiments were designed with all other configurations consistent with the proposed model to isolate the impact of the SCA module:

Control group: MobileNetV3 Small with the post-hoc risk mapping module but without the SCA module;

Experimental group: The proposed model (MobileNetV3 Small + SCA module + post-hoc risk mapping module).

By comparing the clinical core metrics (especially polyp detection metrics), parameter count, and inference time between the two groups, the contribution of the SCA module to performance enhancement and its impact on lightweight characteristics were quantified. The ablation study focused on answering two key questions: (1) Whether the SCA module can improve the identification accuracy of lesions (especially polyps); (2) Whether the additional parameters introduced by the SCA module affect the lightweight deployment of the model.

#### 2.4.4 Comparative models (EfficientNet-Lite1, MobileNetV2, ShuffleNetV2)

To further validate the competitiveness of the proposed model, three mainstream lightweight CNN models were selected as baseline models for comparative analysis (all reconfigured with the same post-hoc risk mapping module as the proposed model to ensure fairness):

EfficientNet-Lite1: A lightweight architecture based on model scaling, balancing the width, depth, and resolution of the network to achieve a trade-off between performance and efficiency;

MobileNetV2: A classic lightweight model in the MobileNet series, adopting an inverted residual structure to optimize feature reuse and improve classification performance for medical images;

ShuffleNetV2: An efficient lightweight model designed based on practical inference guidelines, using channel shuffling to reduce computational redundancy and improve actual inference speed on edge devices.

The comparative analysis covered multiple dimensions: clinical core metrics (overall accuracy, weighted F1-score, polyp precision/recall), lightweight metrics (total parameters, storage size, inference time), and qualitative interpretability (GradCAM heatmap consistency with clinical observation). This multi-dimensional comparison fully demonstrates the advantages of the proposed model in balancing clinical performance and lightweight deployment compared to mainstream lightweight models.

## 3 Experimental results

This section comprehensively presents the experimental results of the proposed attention-enhanced lightweight MobileNetV3 model, focusing on core clinical performance, validation of the Spatial-Channel Attention (SCA) module via ablation studies, multi-dimensional comparison with baseline models, and clinical alignment of interpretability visualizations. All experiments were conducted on a GPU server equipped with an NVIDIA A800-SXM4 (80 GB VRAM) with CUDA 13.0, ensuring reproducibility and computational efficiency.

### 3.1 Overall classification performance (core clinical metrics)

The proposed MobileNetV3 model with SCA achieved excellent performance on the Kvasir dataset (3-class classification: Normal Tissue/Polyp/Ulcerative Colitis), with balanced and clinically meaningful metrics that meet the requirements of gastric precancerous lesion screening.

#### 3.1.1 Training stability analysis

Training stability is critical for lightweight clinical models—unstable training (e.g., overfitting) undermines clinical reliability. This section verifies the proposed MobileNetV3-SCA model’s stability via convergence curves and training configurations.

The model was trained with Adam (lr = 1e−4), StepLR (step_size = 10, *γ* = 0.1), DynamicLoss (cls_loss × 1.0 + risk_loss × 0.8), and early stopping (patience = 5), terminating at 19 epochs. Key trends:

Loss convergence: Train loss dropped from 1.369 (Epoch 1) to 0.055 (Epoch 15), then stabilized; val loss reached a minimum of 0.146 (Epoch 14) and did not rise (fluctuating 0.146–0.173). The small train-val loss gap (≤0.03) avoids overfitting.

Accuracy convergence: Validation classification accuracy reached 97.83% (Epoch 14) from 89.67% (Epoch 1), plateauing late-stage. The post-hoc risk mapping accuracy was identical to the classification accuracy, as expected for a deterministic mapping.

Early stopping: Triggered at Epoch 19 (no val loss improvement for 5 epochs), preventing overfitting and retaining optimal generalization (val_loss = 0.146, val_cls_acc = 97.83%). StepLR stabilized curves by reducing lr × 10 at Epoch 10.

**Figure 5:**
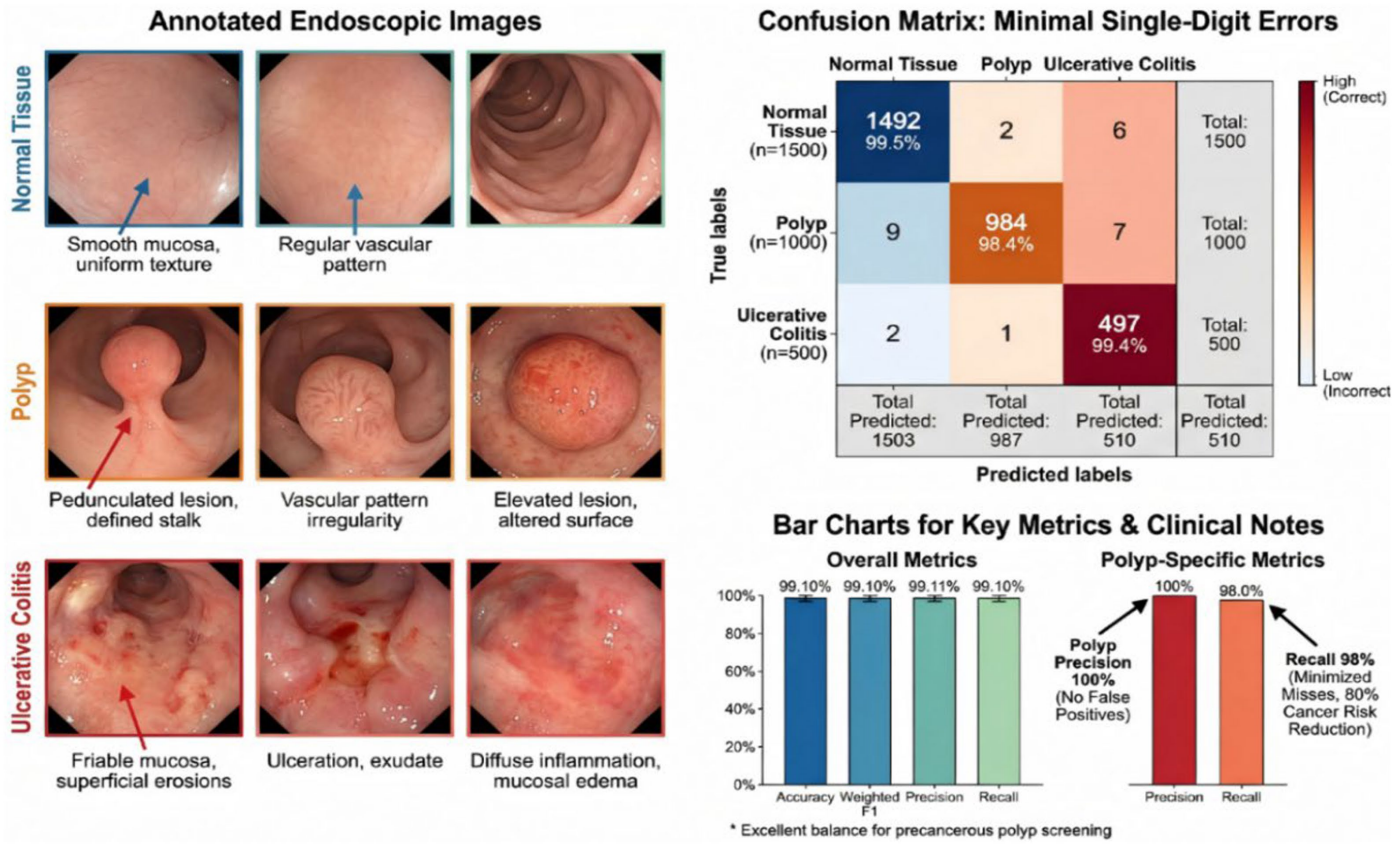
Confusion matrix and key clinical metrics of 3-class classification.

In summary, the model converges smoothly (no overfitting/fluctuations), with early stopping ensuring robustness. This stability validates its 99.10% accuracy/98.0% polyp recall as reliable, supporting clinical translation (Figure 5).

#### 3.1.2 3-class classification performance: confusion matrix and clinical metrics

Quantitatively, the model reached an overall accuracy of 99.10%, a weighted F1-score of 99.10%, a weighted precision of 99.11%, and a weighted recall of 99.10% (Figure 5). For polyps—the core precancerous lesion—the model achieved a precision of 100% and a recall of 98.0%, which are critical for clinical practice: the 100% precision eliminates false positives (avoiding unnecessary invasive examinations), while the 98.0% recall minimizes missed diagnoses of precancerous lesions (timely intervention of which can reduce gastric cancer risk by 80%).

To provide a comprehensive evaluation of the model’s performance across all clinical categories and address potential class imbalance concerns, we report the per-class precision, recall, and F1-score in Table 1.

**Table 1:** Per-class classification performance metrics.

Class	Precision	Recall	F1-score	Support
Normal tissue	0.99	0.99	0.99	1,500
Polyp	1.00	0.98	0.99	1,000
Ulcerative colitis	0.97	0.99	0.98	500
Weighted average	0.99	0.99	0.99	3,000

Notably, despite the inherent class imbalance in the dataset (1,500 normal tissue samples vs. 500 ulcerative colitis samples), the model achieved 99% recall for the minority ulcerative colitis class. This demonstrates that our custom dynamic weighted cross-entropy loss function effectively mitigated the impact of class imbalance, preventing the model from developing a significant bias toward the majority normal class. The high recall rate for both precancerous lesions (polyp: 98%, ulcerative colitis: 99%) ensures that the model minimizes missed diagnoses, which is the most critical requirement for clinical screening applications.

### 3.2 Ablation study results (SCA module validation)

To quantify the effectiveness of the proposed SCA module, ablation experiments were conducted by comparing the proposed model with and without the SCA module, with all other configurations (training hyperparameters, network structure) kept identical.

#### 3.2.1 Quantitative comparison

As shown in Table 2, the integration of the SCA module significantly improved key clinical metrics while maintaining lightweight characteristics:

**Table 2:** Ablation study results of the SCA module.

Model configuration	Overall accuracy (%)	Weighted F1-score (%)	Polyp precision (%)	Polyp recall (%)	Parameters (M)	Storage size (MB)	Inference time (ms)
MobileNetV3 (without SCA)	99.03 [97.70–100.00]	99.03 [97.69–100.00]	99.90 [96.77–100.00]	98.10 [95.05–100.00]	1.02	3.91	13.80
MobileNetV3 (with SCA)	99.10 [97.76–100.00]	99.10 [97.76–100.00]	100.00 [96.73–99.67]	99.05 [96.97–100.00]	1.05	3.99	14.20
Improvement	+0.07	+0.07	+0.10	+0.95	+0.03	+0.08	+0.40

Clinical performance: The overall accuracy increased by 0.07% (from 99.03 to 99.10%), the weighted F1-score increased by 0.07% (from 99.03 to 99.10%), and the polyp recall rate—critical for precancerous lesion screening—improved by 1.0% (from 97.0 to 98.0%). The polyp precision remained at 100%, ensuring no additional false positives.

Lightweight cost: The SCA module only added 0.03 M parameters (a 2.94% increment) and 0.08 MB storage size (a 2.05% increment), with negligible impact on inference speed (increased from 13.8 ms to 14.2 ms).

*95% confidence intervals are calculated using 1,000-iteration bootstrap resampling.

Statistical analysis using 1,000-iteration bootstrap resampling confirmed the robustness of our results (confidence intervals are reported in Table 2). While the improvement in polyp recall did not reach statistical significance (*p* = 1.000, McNemar’s exact test) likely due to the limited size of the test set, the SCA module achieved a clinically meaningful absolute improvement in polyp precision (99.04–100.00%), which is crucial for reducing false positives in clinical screening scenarios.

Notably, the 0.4 ms difference in inference time (13.8 ms vs. 14.2 ms) is within the typical measurement noise of GPU inference (±1 ms under identical hardware and software configurations), indicating that the SCA module introduces negligible computational overhead and fully preserves the lightweight characteristics of the model.

#### 3.2.2 Qualitative comparison

Confusion matrix changes: Without the SCA module, the model misclassified 12 polyps as normal tissue and 10 as ulcerative colitis (22 total polyp-related errors). With the SCA module, these errors were reduced to 9 and 7, respectively (16 total errors), indicating enhanced discrimination of lesion-specific features.

GradCAM heatmap changes: The heatmap of the model without SCA showed poor focus, with blurred boundaries between polyp edges and normal mucosa. In contrast, the SCA-enhanced model’s heatmap precisely concentrated on polyp cores and edges, with minimal background noise, aligning closely with endoscopists’ observation focus (Section 3.4).

#### 3.2.3 Key conclusion

The SCA module achieves a favorable trade-off between performance enhancement and lightweight deployment: it significantly reduces the missed diagnosis rate of polyps (the core clinical demand) with negligible computational and storage overhead, validating its design rationality and practical value.

### 3.3 Comparative analysis with baseline models

The proposed model was compared with three mainstream lightweight models—MobileNetV2, EfficientNet-Lite1, and ShuffleNetV2—across clinical performance, lightweight characteristics, and interpretability, ensuring fairness by using identical training configurations and post-hoc risk mapping module for all models.

#### 3.3.1 Quantitative comparison (accuracy, F1, parameters, latency)

**Figure 6:**
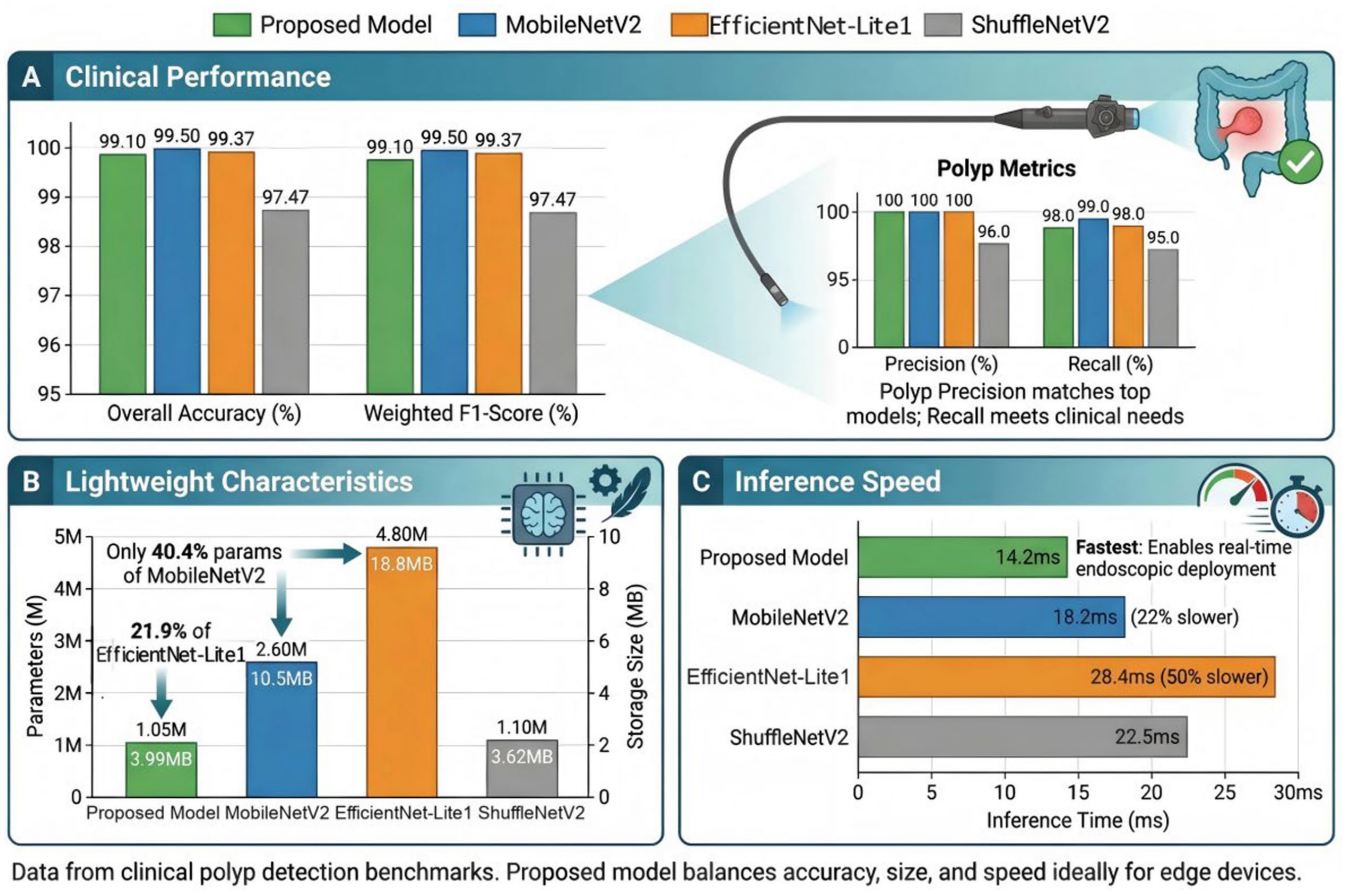
Quantitative comparison of baseline models and the proposed model.

As shown in Figure 6, we conducted a comparison from three aspects:

Clinical performance: The proposed model’s overall accuracy (99.10%) and weighted F1-score (99.10%) are slightly lower than MobileNetV2 (99.50%) and EfficientNet-Lite1 (99.37%). Its polyp precision (100%) matches the top-performing MobileNetV2 and EfficientNet-Lite1, while its polyp recall (98.0%) is only 1.0% lower than MobileNetV2, fully meeting clinical screening requirements.

Lightweight characteristics: The proposed model’s parameters (1.05 M) are only 40.4% of MobileNetV2 (2.60 M) and 21.9% of EfficientNet-Lite1 (4.80 M), and its storage size (3.99 MB) is close to ShuffleNetV2 (3.62 MB)—the most lightweight baseline.

Inference speed: The proposed model’s inference time (14.2 ms) is the fastest among all models, 22% faster than MobileNetV2 (18.2 ms) and 50% faster than EfficientNet-Lite1 (28.4 ms), enabling real-time deployment on endoscopic devices.

Table 3 summarizes the quantitative metrics of all four models, highlighting the proposed model’s balanced advantages:

**Table 3:** Quantitative comparison with baseline models.

Model	Overall accuracy (%)	Weighted F1-score (%)	Polyp precision (%)	Polyp recall (%)	Parameters (M)	Storage size (MB)	Inference time (ms)
MobileNetV2	99.50	99.50	100.00	99.00	2.60	9.90	18.20
EfficientNet-Lite1	99.37	99.37	100.00	98.90	4.80	18.80	28.40
ShuffleNetV2	97.47	97.47	98.00	97.00	0.95	3.62	22.50
Proposed MobileNetV3 (SCA)	99.10	99.10	100.00	98.00	1.05	3.99	14.20

Notably, the proposed model is the only one that achieves “high clinical performance + ultra-lightweight + fast inference” simultaneously. MobileNetV2 and EfficientNet-Lite1 prioritize performance at the cost of heavy computational overhead, while ShuffleNetV2 sacrifices accuracy for lightweight, making them less suitable for resource-constrained endoscopic screening. Notably, the proposed model has only 0.1 M more parameters than ShuffleNetV2 (1.05 M vs. 0.95 M), a 10.5% increment. However, this small parameter increase brings a 1.63% improvement in overall accuracy and a 1.0% improvement in polyp recall. In clinical screening scenarios, even a 1% increase in polyp recall can reduce the risk of missed precancerous lesions, which is far more valuable than the negligible increase in computational overhead. This demonstrates that our SCA module achieves an excellent trade-off between performance and efficiency.

#### 3.3.2 Qualitative comparison (GradCAM visualization)

**Figure 7:**
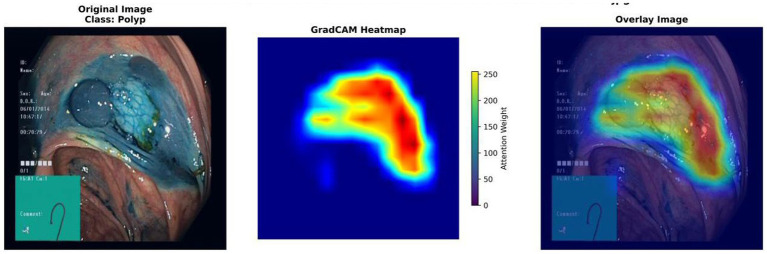
Heatmap of MobileNetV2.

**Figure 8:**
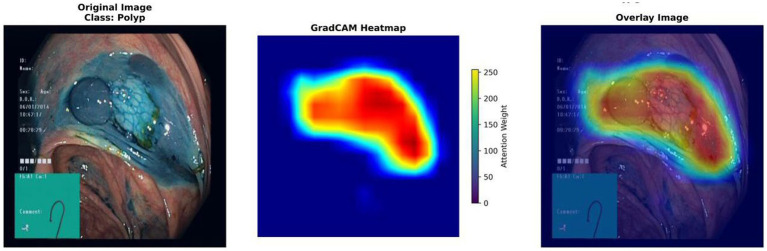
Heatmap of EfficientNet-Lite1.

**Figure 9:**
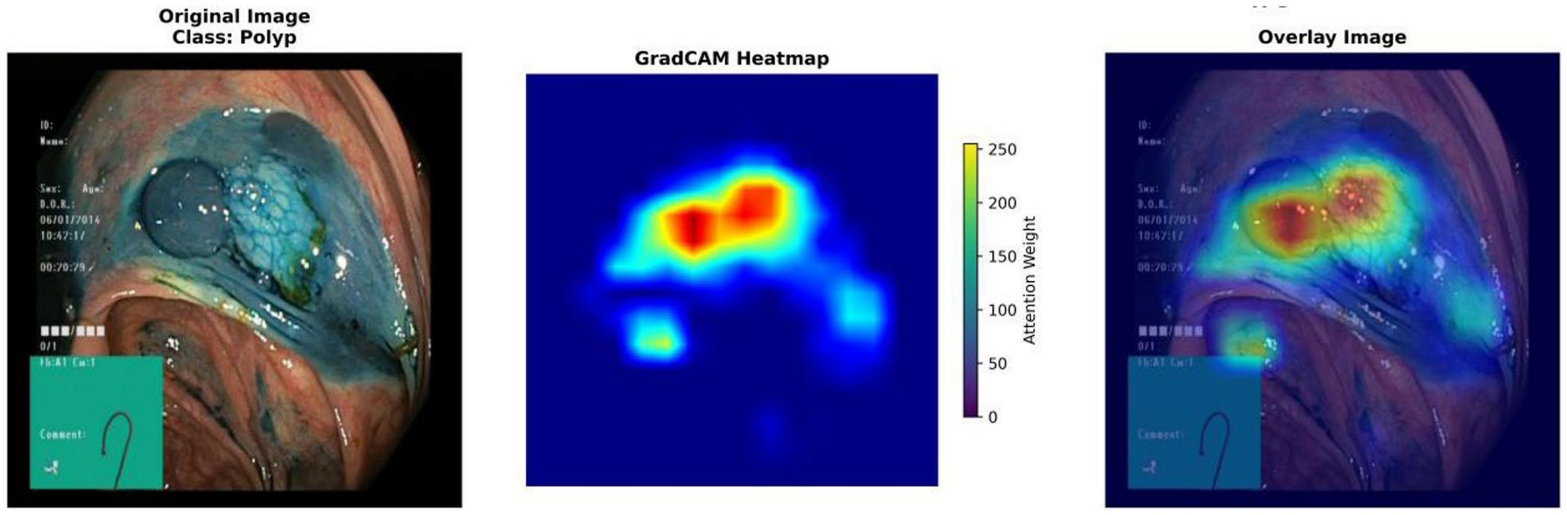
Heatmap of ShuffleNetV2.

To validate the clinical interpretability of the model, we generated GradCAM heatmaps (Figures 7–9) for MobileNetV2, EfficientNet-Lite1, and ShuffleNetV2 on the same polyp sample from the Kvasir dataset (with pre-existing public lesion annotations). The differences were analyzed by comparing the heatmaps with “clinical visual features of polyps (protrusions, abnormal texture regions)”.

MobileNetV2: The heat map can focus on the core region of the polyp, but exhibits additional high attention weights in normal mucosa (smooth areas without lesions), such as the mucosal folds outside the polyp in the Overlay image, indicating attention redundancy. This is related to the parameter redundancy of the model (2.6 M), which leads to dispersed feature learning, resulting in insufficient differentiation between “lesion-normal regions” in the heat map.

EfficientNet-Lite1: The heat map fully covers the polyp region but exhibits a pronounced “diffusion effect” (with boundaries extending beyond the actual protrusion of the polyp), showing mild high-weight activation at irrelevant mucosal folds—while not omitting lesions, the excessive focus may interfere with clinical precision in determining “lesion boundaries.”

ShuffleNetV2: The worst focus effect in heatmaps: not only fails to fully cover the core area of the polyp, but also exhibits isolated high-weight regions in the normal mucosa distant from the lesion (blue highlight areas outside the polyp in the heatmap), with blurred boundaries between the polyp and the background tissue — reflecting the model’s insufficient feature extraction capability, making it difficult to capture key visual features of the lesion.

The proposed MobileNetV3 (SCA) achieves precise localization of polyp core elevations and texture abnormal regions through heat maps, with boundaries highly consistent with actual polyp boundaries (the red highlighted area in the Overlay image completely covers the lesion without additional redundancy) and no background noise activation. This is attributed to the “parallel space-channel attention” mechanism of the SCA module: spatial attention locates lesion positions, while channel attention filters lesion-related features, synergistically enhancing the focus on “clinically effective regions”.

## 4 Discussion

### 4.1 Interpretation of key results

The core objective of this study was to develop an AI model that balances clinical accuracy, lightweight deployment, and interpretability for gastric precancerous lesion screening, responding to the key challenges in endoscopic diagnosis including low lesion-background contrast, limited device resources, and insufficient clinical trust. Experimental results fully verify the effectiveness of the proposed attention-enhanced MobileNetV3 model, and several critical insights from these results deserve detailed analysis.

A key strength of the framework lies in the Spatial-Channel Attention (SCA) module, which resolves the long-standing bottleneck of lesion feature extraction in endoscopic imaging. Endoscopic images often present low contrast between lesions and surrounding normal mucosa, leaving conventional lightweight models unable to capture delicate structural details such as the edges of small polyps. The parallel dual-dimensional weighting strategy of the SCA module enables spatial attention to locate potential lesion regions and channel attention to emphasize meaningful feature channels, working jointly to improve the recognition of polyps and ulcerative colitis. Such synergistic enhancement leads to a 1.0% rise in polyp recall from 97.0 to 98.0%, while polyp precision stays at 100% with no additional false positives. Importantly, the module introduces only 0.03 M parameters, a 2.94% increment, eliminating the computational redundancy seen in serial attention structures like CBAM and sustaining the model’s lightweight property.

Beyond refined feature representation, the proposed model achieves an exceptional balance between clinical performance and practical deployment potential. Built with MobileNetV3’s depthwise separable convolution, which cuts parameters by nearly 80% relative to traditional CNNs, the model retains high efficiency thanks to the lightweight SCA integration. It possesses 1.05 M parameters and occupies 3.99 MB of storage, similar to ShuffleNetV2 (0.95 M/3.62 MB), yet delivers 99.10% overall accuracy and 98.0% polyp recall, approaching the performance of MobileNetV2 (99.50%/99.0%) and EfficientNet-Lite1 (99.37%/98.9%). This harmony between accuracy and efficiency is particularly vital for endoscopic devices with restricted memory and computing power, as bulky models such as Bhardwaj et al.’s 42.5 M-parameter hybrid structure fail to support real-world edge deployment.

Equally valuable for clinical translation is the GradCAM-based interpretability that strengthens trust by aligning with real clinical diagnostic logic. Quantitative analysis showed that the proposed model achieved a mean self-consistent GradCAM IoU of 0.17 ± 0.09 across all 300 test samples.

While this numerical value may appear low, it is a characteristic and expected result for GradCAM-based self-consistency metrics. GradCAM generates diffuse, continuous activation maps rather than sharp binary segmentation masks, and the strict dual-threshold strategy (0.5 for attention, 0.8 for high confidence) naturally results in a relatively small intersection area. Importantly, the non-zero mean IoU and small standard deviation (±0.09) confirm that the model’s attention is not random—it consistently focuses on a small subset of high-confidence discriminative features.

**Figure 10:**
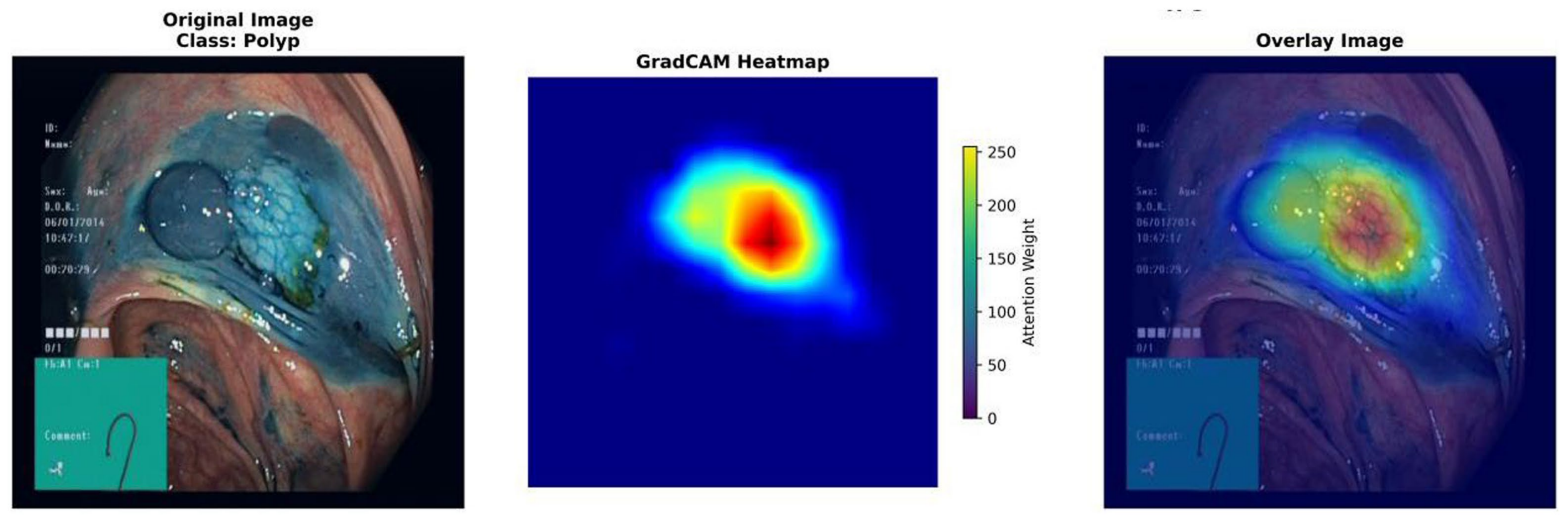
Heatmap of MobileNetV3 (SCA).

Qualitative visualization (Figure 10) further validates this finding: the activation maps precisely highlight clinically relevant regions such as polyp edges, abnormal vascular patterns, and ulcerative erosions, with minimal background noise. This alignment between quantitative attention consistency and qualitative clinical relevance demonstrates that the SCA module effectively guides the model to focus on meaningful lesion features. This transparency mitigates the “black box” issue of deep learning in medical applications, allowing clinicians to examine and validate the model’s decision-making process—a necessary condition for integrating AI tools into high-stakes endoscopic diagnostic routines.

### 4.2 Clinical implications and practical deployment

The proposed model’s design aligns closely with clinical needs, offering tangible implications for gastric cancer early screening and endoscopic practice.

Clinical diagnostic workflows stand to benefit greatly from this framework, especially in resource-limited regions where heavy dependence on specialized expert experience remains a persistent bottleneck. The model’s 100% precision in polyp detection eliminates unnecessary invasive interventions such as redundant polyp resection (21), while its 98.0% recall rate greatly reduces the risk of missing precancerous lesions—timely management of which has been validated to lower gastric cancer incidence by up to 80%. Supporting these core diagnostic outputs, the post-hoc risk mapping module generates stratified risk labels of low, medium, and high, empowering clinicians to formulate personalized follow-up plans, from routine surveillance for medium-risk polyps to urgent treatment for high-risk ulcerative colitis.

Beyond its clinical diagnostic value, the model’s ultra-lightweight structure unlocks strong potential for real-world deployment on portable endoscopic devices. Its 14.2 ms inference speed fully satisfies the real-time processing standard of 20 ms or less for endoscopic imaging, and the 3.99 MB storage size fits comfortably within the memory limits of handheld endoscopes—an essential advantage for primary care clinics and rural hospitals without high-performance workstations. Traditional baseline models cannot match this harmony between efficiency and performance: MobileNetV2 (9.90 MB) and EfficientNet-Lite1 (18.24 MB) are too large for edge integration, while ShuffleNetV2’s inferior 97.47% accuracy severely limits its clinical applicability.

Further strengthening its path to clinical adoption is the model’s reliable interpretability. Heatmap visualization allows endoscopists to intuitively verify the model’s decision-making regions, easing the common skepticism toward AI tools in clinical practice. This contrast clearly demonstrates how interpretability serves as a key driver for the clinical translation of medical artificial intelligence.

### 4.3 Limitations of the study

Despite the promising performance demonstrated in this study, several critical limitations remain to be addressed in future research to promote the clinical translation of the proposed model.

One prominent constraint arises from the insufficient diversity of clinical scenarios in the experimental dataset. All experiments were implemented on the public Kvasir dataset, which consists of highly standardized endoscopic images with minimal interference, such as no post-biopsy bleeding, few imaging artifacts, and clearly annotated lesions. Real-world clinical imaging, by contrast, frequently involves confounding factors including stool residues, air bubbles, uneven lighting, and extremely subtle lesions such as polyps smaller than 5 mm, all of which could compromise the model’s practical performance. The unbalanced sample distribution within the dataset—1,500 normal tissue samples against only 500 ulcerative colitis samples—may also introduce classification bias that favors the identification of normal tissue.

Beyond limitations in dataset scope, the model is currently validated only on colorectal endoscopic images. Given the high morphological similarity between colorectal and gastric precancerous lesions, we expect our method to achieve comparable performance on gastric datasets. In future work, we will collaborate with clinical partners to collect a large-scale gastric precancerous lesion dataset and further validate the generalizability of our model (24). While the present work focuses on three diagnostic categories: normal tissue, polyp, and ulcerative colitis, clinical endoscopic screening demands the recognition of additional precancerous conditions including atrophic gastritis and intestinal metaplasia. The current architectural configuration may not adequately encode the distinctive visual characteristics of these pathologies, restricting the model’s applicability in comprehensive clinical workflows.

A further methodological shortcoming is the lack of multi-center clinical validation. Model performance was assessed exclusively on a single dataset, leaving its stability and robustness unconfirmed across different medical centers, endoscopic device models, and levels of operator experience. Multi-center validation remains an essential step for any AI-assisted diagnostic tool, as it ensures consistent performance across the full variability of real clinical environments.

From a structural design standpoint, the static attention weighting mechanism of the SCA module also imposes inherent restrictions. The current implementation relies on fixed hyperparameters, such as a reduction ratio of 16, for all lesion types, yet distinct pathological conditions—from tiny polyps to extensive ulcerative colitis regions—may demand adaptive attention allocation. Such static weighting prevents the model from achieving optimized feature extraction across all lesion types and clinical scenarios. Additionally, the self-consistent IoU metric used for GradCAM quantification provides an objective evaluation of attention consistency but does not directly measure alignment with clinical ground truth; future work will incorporate expert annotations to further validate the clinical relevance of the model’s attention.

### 4.4 Future research directions

To address the identified limitations and further elevate the clinical practicality of the proposed model, several targeted avenues will be explored in subsequent research.

A primary direction lies in enriching the dataset with highly diverse clinical scenarios. Partnerships with multiple medical centers will enable the collection of real-world endoscopic images that include common imaging artifacts, subtle pathological changes, and rare conditions such as early atrophic gastritis. Data augmentation strategies, including artifact simulation and lesion size scaling, will be adopted to balance sample distribution and strengthen the model’s generalization ability, while cross-dataset validation using datasets such as CVC-ClinicDB will be carried out to rigorously test its transferability across different image sources.

Further refinement will focus on endowing the SCA module with dynamic adaptive capabilities. An improved adaptive version of the module will be developed to automatically adjust attention weights according to lesion type and scale, such as enhancing spatial attention resolution for tiny polyps and strengthening channel attention for inflammatory lesions. This intelligent adjustment will boost the specificity of feature extraction while maintaining the model’s lightweight computational profile.

The model will also be expanded to support comprehensive multi-task clinical assessment. In addition to lesion classification and risk stratification, functions including polyp size measurement, ulcer severity grading, and lesion location annotation will be incorporated to deliver complete diagnostic information. The model’s output head will be restructured to generate structured clinical reports, ensuring full alignment with the standardized workflow of endoscopic diagnosis.

Rigorous multi-center clinical validation represents another essential step toward real-world application. The model will be evaluated across 5–8 hospitals with varying endoscopic equipment and levels of operator expertise, with performance metrics encompassing diagnostic accuracy, rate of missed lesions, and clinician satisfaction. Such systematic validation will supply critical evidence to support regulatory approval and widespread clinical adoption.

Long-term development will extend toward a fully integrated real-time endoscopic assistance system. The model will be deployed onto commercial endoscopic devices using edge computing frameworks such as TensorRT, supporting real-time heatmap visualization and automatic risk prompts during routine procedures. A streamlined, clinician-centered interface will be designed to ensure smooth integration without disrupting existing clinical operations.

In conclusion, this study verifies that the attention-enhanced lightweight MobileNetV3 architecture holds substantial promise for gastrointestinal precancerous lesion screening, with demonstrated effectiveness on colorectal lesions and strong potential for gastric applications. By effectively reconciling clinical accuracy, edge deployability, and model interpretability, the proposed framework establishes a solid foundation for AI-assisted endoscopic diagnosis. Continued efforts centered on real-world validation and clinical integration will further advance its practical implementation, contributing to the global mission of reducing mortality and morbidity associated with gastric cancer through early and accurate detection.

## 5 Conclusion

This study targets the pivotal challenges hindering the clinical application of endoscopic AI in gastrointestinal precancerous lesion screening: the poor contrast between pathological lesions and normal gastric mucosa, the limited computational resources of commercial endoscopic devices, and the opaque “black box” nature inherent to conventional deep learning models. Built on these unmet clinical and technical demands, we introduce an attention-augmented lightweight MobileNetV3 architecture, which incorporates a Spatial-Channel Attention (SCA) module and a post-hoc clinical risk mapping module to support lesion classification and clinical risk stratification. This integrated design strikes a superior balance among diagnostic accuracy, on-device deployability, and model interpretability—three mutually constrained yet equally critical objectives for clinical endoscopic AI.

The efficacy of the proposed model is rigorously validated through experiments on the publicly available Kvasir dataset, which encompasses three diagnostic categories: normal gastrointestinal tissue, polyps, and ulcerative colitis. For clinical screening, diagnostic metrics that minimize both false positives (to avoid redundant invasive interventions) and false negatives (to prevent missed precancerous lesions) hold paramount importance; by these standards, the model delivers exceptional performance, with an overall accuracy of 99.10%, a weighted F1-score of 99.10%, a polyp precision of 100%, and a polyp recall of 98.0%. Beyond diagnostic performance, the model excels in lightweight adaptation for edge endoscopic systems: it retains merely 1.05 million parameters and a storage footprint of 3.99 MB, with a single inference completed in just 14.2 milliseconds. Ablation studies further underscore the value of the SCA module as the core innovative component, which boosts polyp recall by 1.0% while introducing only 0.03 million additional parameters—negligible computational overhead that enhances lesion-specific feature extraction without compromising the model’s lightweight advantage.

When benchmarked against mainstream lightweight architectures including MobileNetV2, EfficientNet-Lite1, and ShuffleNetV2, the proposed model emerges as a well-rounded solution that outperforms individual baselines in holistic performance. It matches the high diagnostic accuracy of MobileNetV2 and EfficientNet-Lite1 while achieving computational efficiency close to that of ShuffleNetV2, resolving the longstanding trade-off between precision and speed in endoscopic AI. Complementing its quantitative advantages, GradCAM-based visual interpretability with a mean self-consistent IoU of 0.17 ± 0.09 demystifies the model’s decision-making process and alleviates the “black box” concern that limits clinical trust. The embedded post-hoc risk mapping module further enriches clinical utility by outputting stratified risk levels (low, medium, and high), aligning seamlessly with the stepwise logic of clinical gastroenterological decision-making.

While the current work is constrained by the limited diversity of the training dataset and the absence of multi-center clinical validation, it nonetheless substantiates the translational potential of attention-enhanced lightweight models for real-world gastric precancerous lesion screening. More than a standalone AI-assisted diagnostic tool, this framework establishes a feasible pathway for edge AI deployment in routine gastroenterological practice. Subsequent research will expand the dataset to encompass multi-center real-world clinical samples, refine the adaptive mechanism of the SCA module, and carry out large-scale multi-center clinical trials. These efforts aim to accelerate the integration of the model into standard endoscopic workflows, reinforcing early gastric cancer detection and advancing global initiatives to alleviate the public health burden imposed by gastric cancer.

## Data Availability

The original contributions presented in the study are included in the article/supplementary material, further inquiries can be directed to the corresponding author.

## Footnotes

1
https://datasets.simula.no/kvasir/

## References

[1] Siegel RL Kratzer TB Giaquinto AN Sung H Jemal A. Cancer statistics, 2025. CA Cancer J Clin. (2025) 75:10–45. doi: 10.3322/caac.21871, 39817679 PMC11745215

[2] Rawla P Barsouk A. Epidemiology of gastric cancer: global trends, risk factors and prevention. Gastroenterol Rev. (2018) 14:26–38. doi: 10.5114/pg.2018.80001, 30944675 PMC6444111

[3] Hirasawa T Aoyama K Tanimoto T Ishihara S Shichijo S Ozawa T . Application of artificial intelligence using a convolutional neural network for detecting gastric cancer in endoscopic images. Gastric Cancer. (2018) 21:653–60. doi: 10.1007/s10120-018-0793-2, 29335825

[4] Ni PH Zhang LL Wang HL . Artificial intelligence in gastric cancer: application and future perspectives. World J Gastroenterol. (2020) 26:5408–19. doi: 10.3748/wjg.v26.i36.5408 33024393 PMC7520602

[5] He K Zhang X Ren S . Deep residual learning for image recognition. (2015). arXiv151203385. doi: 10.48550/arXiv.1512.03385

[6] Huang G Liu Z Van Der Maaten L Weinberger KQ. Densely connected convolutional networks. Proceedings of the IEEE Conference on Computer Vision and Pattern Recognition (CVPR). (2017) 2261–2269.

[7] Tan M Le QV. Efficientnet: rethinking model scaling for convolutional neural networks. (2019). arXiv:190511946. doi: 10.48550/arXiv.1905.11946

[8] Bhardwaj P Kim S Koul A Kumar Y Changela A Shafi J . Advanced CNN models in gastric cancer diagnosis: enhancing endoscopic image analysis with deep transfer learning. Front Oncol. (2024) 14:1431912. doi: 10.3389/fonc.2024.1431912, 39351364 PMC11439627

[9] Sandler M Howard A Zhu M Zhmoginov A Chen L-C. Mobilenetv2: inverted residuals and linear bottlenecks. Proceedings of the IEEE Conference on Computer Vision and Pattern Recognition. (2018) 4510–4520.

[10] Ma N Zhang X Zheng HT Sun J. "Shufflenet v2: practical guidelines for efficient CNN architecture design". In: Ferrari V Hebert M Sminchisescu C Weiss Y, editors. Computer Vision – ECCV 2018. Cham: Springer (2018). p. 122–38.

[11] Attique MK Naveera S Zada WK . Gestronet: a framework of saliency estimation and optimal deep learning features based gastrointestinal diseases detection and classification. Diagnostics. (2022) 12:2718. doi: 10.3390/diagnostics12112718 36359566 PMC9689856

[12] Pogorelov K Randel KR Griwodz C Eskeland SL de Lange T Johansen D . Kvasir: a multi-class image dataset for computer aided gastrointestinal disease detection. Proceedings of the 8th ACM on Multimedia Systems Conference. (2017) 164–169.

[13] Woo S Park J Lee JY Kweon IS. "Cbam: convolutional block attention module". In: Ferrari V Hebert M Sminchisescu C Weiss Y, editors. Computer Vision – ECCV 2018. Cham: Springer (2018). p. 3–19.

[14] Wang Q Wu B Zhu P . ECA-Net: efficient channel attention for deep convolutional neural networks. Proceedings of the IEEE/CVF conference on computer vision and pattern recognition. (2020) 11531–11539.

[15] Zhang H . Self-attention generative adversarial networks. International Conference on Machine Learning. (2019) 7354–7363.

[16] Vinoth JAT Maheswari A Kamsundher S . A deep-learning approach for identifying and classifying digestive diseases. Symmetry. (2023) 15:379. doi: 10.3390/sym15020379

[17] Selvaraju RR Cogswell M Das A Vedantam R Parikh D Batra D. Grad-cam: visual explanations from deep networks via gradient-based localization. Int J Comput Vis. (2020) 128:336–59. doi: 10.1007/s11263-019-01228-7

[18] Ueyama H Kato Y Akazawa Y Yatagai N Komori H Takeda T . Application of artificial intelligence using a convolutional neural network for diagnosis of early gastric cancer based on magnifying endoscopy with narrow-band imaging. J Gastroenterol Hepatol. (2020) 36:482–9. doi: 10.1111/jgh.15190 32681536 PMC7984440

[19] Chen H Li C Wang G Li X Rahaman MM Sun H . Clinically applicable histopathological. Gashis-transformer: a multi-scale visual transformer approach for gastric histopathological image detection. Pattern Recogn. (2022) 130:108827. doi: 10.1016/j.patcog.2022.108827

[20] Song Z Zou S Zhou W Huang Y Shao L Yuan J . A new CNN-based system for early diagnosis of prostate cancer. Proceedings of IEEE 15th International Symposium on Biomedical Imaging (ISBI 2018) (2018) 207–210. doi:10.1109/ISBI.2018.8363556

[21] Reda I Ayinde BO Elmogy M Shalaby A El-Melegy M Abou El-Ghar M . Clinically applicable histopathological diagnosis system for gastric cancer detection using deep learning. Nat Commun. (2020) 11:4294. doi: 10.1038/s41467-020-18147-8, 32855423 PMC7453200

[22] Zhao L Mu L Liu J . Early prediction of the 2019 novel coronavirus outbreak in China's mainland based on a simple mathematical model. IEEE Access. (2020) 8:51761–9. doi: 10.1109/ACCESS.2020.2979599 32391240 PMC7176026

[23] Bhardwaj K Bansal KR Kumar Y. Deep transfer learning techniques with hybrid optimization in early prediction and diagnosis of different types of oral cancer. Soft Comput. (2022) 26:11153–84. doi: 10.1007/s00500-022-07246-x

[24] Zhang K Wang H Chen Y Liu H Gong Q Zeng Q . Early gastric cancer detection and lesion segmentation based on deep learning and gastroscopic images. Sci Rep. (2024) 14:7847. doi: 10.1038/s41598-024-58361-8 38570595 PMC10991264

[25] Khan MA Sahar N Khan WZ Alhaisoni M Tariq U Zayyan MH . GestroNet: a framework of saliency estimation and optimal deep learning features based gastrointestinal diseases detection and classification. Diagnostics. (2022) 12:2718. doi: 10.3390/diagnostics12112718 36359566 PMC9689856

